# Restoration of Motion Blurred Image by Modified DeblurGAN for Enhancing the Accuracies of Finger-Vein Recognition

**DOI:** 10.3390/s21144635

**Published:** 2021-07-06

**Authors:** Jiho Choi, Jin Seong Hong, Muhammad Owais, Seung Gu Kim, Kang Ryoung Park

**Affiliations:** Division of Electronics and Electrical Engineering, Dongguk University, 30 Pildong-ro 1-gil, Jung-gu, Seoul 04620, Korea; choijh1027@dongguk.edu (J.C.); turtle1990@dgu.ac.kr (J.S.H.); owais2018@dongguk.edu (M.O.); ismysg104@dgu.ac.kr (S.G.K.)

**Keywords:** Finger-vein recognition, motion blur image restoration, modified DeblurGAN, CNN

## Abstract

Among many available biometrics identification methods, finger-vein recognition has an advantage that is difficult to counterfeit, as finger veins are located under the skin, and high user convenience as a non-invasive image capturing device is used for recognition. However, blurring can occur when acquiring finger-vein images, and such blur can be mainly categorized into three types. First, skin scattering blur due to light scattering in the skin layer; second, optical blur occurs due to lens focus mismatching; and third, motion blur exists due to finger movements. Blurred images generated in these kinds of blur can significantly reduce finger-vein recognition performance. Therefore, restoration of blurred finger-vein images is necessary. Most of the previous studies have addressed the restoration method of skin scattering blurred images and some of the studies have addressed the restoration method of optically blurred images. However, there has been no research on restoration methods of motion blurred finger-vein images that can occur in actual environments. To address this problem, this study proposes a new method for improving the finger-vein recognition performance by restoring motion blurred finger-vein images using a modified deblur generative adversarial network (modified DeblurGAN). Based on an experiment conducted using two open databases, the Shandong University homologous multi-modal traits (SDUMLA-HMT) finger-vein database and Hong Kong Polytechnic University finger-image database version 1, the proposed method demonstrates outstanding performance that is better than those obtained using state-of-the-art methods.

## 1. Introduction

There are several types of measurable human biometrics, including those of voice, face, iris, fingerprint, palm print, and finger-vein recognition. Among these, finger-vein recognition has the following advantages, (1) finger-vein patterns are hidden under the skin. Therefore, they are generally invisible, making them difficult to forge or steal. (2) Non-invasive image capture ensures both convenience and cleanliness and is more suitable for a user. (3) As most people have ten fingers, if an unexpected accident occurs with one finger, the other finger can be used for authentication [1]. However, due to various factors such as light scattering in the skin layer caused by near-infrared (NIR) light, focus mismatch of a camera lens, differences in finger thickness, differences in depth between the surface of the skin and vein, and finger movements, blurring may occur when capturing finger-vein images. Blurred images generated in these kinds of blur can significantly reduce finger-vein recognition performance. Therefore, image restoration through a deblurring method is necessary. Extensive research has been conducted for restoring skin scattering blur that occurs frequently [2,3,4,5,6,7,8,9], and several studies have been conducted on optical blur caused by the difference in the distance from a camera lens to the finger vein and finger thickness [10,11]. Motion blur can occur frequently, due to finger movement. However, no study has been conducted for motion blurred finger-vein image restoration.

Although, during finger-vein image capture, a finger is fixed to the image capturing device to some extent; however, Parkinson’s disease, physiologic tremors, dystonia, and excessive stress may cause hand tremors. Due to these reasons, motion blur can occur. Furthermore, with the recent expansion of non-contact devices due to COVID-19, motion blur occurs more in the input image, and the resulting motion blurred image causes severe performance degradation during finger-vein recognition. To solve this problem, the restoration of a motion blurred finger-vein image is essential.

Conventional image restoration methods can be categorized into blind and non-blind deblurring [12]. Early non-blind deblurring methods perform deblurring, assuming that blur kernels are known. Blur kernel is deduced from knowledge of the image formation process (e.g., amount of motion or defocus blur and camera sensor optics), calculated from the test image, or measured through point spread function (PSF) [13]. Using these methods, the original sharp image can be obtained through deconvolution by estimating the blur kernel. However, when a non-blind restoration method is applied, the recognition performance can be reduced if images are acquired from various devices and show difference blurring characteristics in the spatial domain. Moreover, there are limitations to applying non-blinded methods to each case because various types of distortions occur when capturing an image in actual environments. Also, most blur kernels are unknown in actual environments, and it is time-consuming to estimate blur kernels.

Contrary to the non-blind deblurring method, the blind deblurring methods proceed with deblurring, assuming that blur kernels are unknown [12,14,15]. A generative adversarial network (GAN) that combines the blind deblurring method and the training-based method has also been studied to solve the problems arising from non-blinded deblurring [12,14]. GAN is a network that generates an image by finding an optimal filter using weights trained from the training data. Therefore, using GAN has the advantage of being robust even if images have various distortions. Also, there is no need to estimate the blur kernel directly, and restoration can be performed through training. Considering these reasons, we propose a method of performing motion blurred finger-vein image restoration using the newly proposed modified DeblurGAN and a method of performing restored finger-vein image recognition using deep CNN. The main contributions of our paper are as follows:

- This is the first study on motion blur finger-vein image restoration that can occur in actual environments.
- For restoration of motion blur finger-vein image, we propose a modified DeblurGAN. The proposed modified DeblurGAN has differences in comparison with the original DeblurGAN, (1) dropout layer removal, (2) number of trainable parameters reduction by modifying the number of the residual block structure, (3) and uses feature-based perceptual loss in the first residual block.
- Training is conducted by separating the modified DeblurGAN and the deep CNN, therefore, reducing training complexity while improving convergence.
- The modified DeblurGAN, a deep CNN, and a non-uniform motion blurred image database are published in [16] to allow other researchers to perform fair performance evaluations.

This paper is organized as follows: Section 2 provides an overview of the previous studies, and the proposed method is explained in Section 3. In Section 4, comparative experiments and experimental results with analysis are described. Finally, in Section 5, the conclusions of this paper are explained.

## 2. Related Works

Previous studies on blurred finger-vein image restoration have been conducted on the restoration of skin scattering or optical blur, and studies related to motion blur restoration have not been conducted. Therefore, previous studies were analyzed in terms of finger-vein recognition without blur restoration, with skin scattering blur restoration, and with optical blur restoration. Such methods can be further categorized into handcrafted feature-based and deep-feature-based finger-vein recognition for analysis.

### 2.1. Finger-Vein Recognition without Blur Restoration

For the handcrafted feature-based finger-vein recognition without blur restoration method, Lee et al. [17] proposed a method for finger-vein recognition by aligning the image using minutia points extracted from the finger-vein region, extracting finger-vein features using a local binary pattern (LBP), and calculating the Hamming distance using the extracted features. Peng et al. [18] applied Gabor filters having eight orientations to the original finger-vein image and extracted the finger-vein pattern by the fusion of the image with the vein pattern highlighted. They proposed a scale-invariant feature transform (SIFT) feature matching method based on the extracted finger-vein patterns. The method proposed in the study of [18] has the advantage that recognition performance is improved when an optimal filter is accurately modeled. However, this method can cause performance degradation when the filter is applied to finger-vein images having multiple characteristics, and since this experiment was conducted in a constraint environment, it is not robust to image variants, such as illumination or misalignment. Moreover, they did not consider the blur that could occur when capturing a finger-vein image.

Deep feature-based methods have been studied to overcome the drawbacks of these handcrafted feature-based methods. Although a deep-learning-based method was not used, Wu et al. [19] performed dimension reduction and feature extraction of a finger-vein image using a principal component analysis (PCA) and a linear discriminant analysis (LDA). They proposed a finger-vein pattern identification method based on a support vector machine (SVM), which used the PCA- and LDA-extracted features. Hong et al. [20] and Kim et al. [21] proposed finger-vein verification methods to distinguish genuine (authentic) matching (matching images of the same class), and imposter matching (matching images of different classes) using the difference image of enrolled and input images as input to a CNN. Qin et al. [22] created vein-pattern maps, calculated the finger-vein feature probability for each pixel, and labeled veins and backgrounds. Subsequently, training was conducted by dividing the original image into an N×N size, and the probability that the final input image was the vein pattern was calculated. Song et al. [23] and Noh et al. [24,25] proposed a shift-matching finger-vein recognition method using a composite image. Qin et al. [26] proposed a finger-vein verification method that combined a CNN and long short-term memory (LSTM). They assigned labels through handcrafted finger-vein image segmentation techniques and extracted finger-vein features using stacked convolutional neural networks and long short-term memory (SCNN-LSTM). Genuine and imposter matching were verified using feature matching between supervised feature encoding and enrollment databases using extracted features. These studies on deep feature-based finger-vein recognition have a limitation that an intensive training process is required, and there is a disadvantage that they did not consider blur that can occur when capturing finger-vein images.

### 2.2. Finger-Vein Recognition with Skin Scattering Blur Restoration

Lee et al. [2] proposed a method for restoring skin scattering blur by measuring a PSF of a skin scattering blur and using a constrained least squares (CLS) filter. Yang et al. [3,4] performed scattering-removal by calculating light-scattering components of a biological optical model (BOM). Yang et al. [5] performed scattering effects removal from finger-vein images by considering an anisotropic diffusion, and gamma correction (ADAGC), weighted biological optical model (WBOM), Gabor wavelet, non-scattered transmission map (NSTM), and inter-scale multiplication operation. Shi et al. [6] used haze-removal techniques based on Koschmieder’s law to remove scattering effects in finger-vein images. Yang et al. [7] used multilayered PSF and BOM to restore blurred images. Furthermore, Yang et al. [8] proposed a scattering-effect removal method using a BOM-based algorithm that measured the scattering component with the transmission map. You et al. [9] designed a bilayer diffusion model to simulate light scattering and measured the parameters of a bilayer diffusion model through blur-Steins unbiased risk estimate (blur-SURE). Image restoration methods were also proposed based on these parameters with the multi-Wiener linear expansion thresholds (SURE-LET). However, these studies have the disadvantages that scattering blur parameters must be accurately estimated, and parameters must be re-estimated when the domain between the image used for estimation and the test image is different.

### 2.3. Finger-Vein Recognition with Optical Blur Restoration

Lee et al. [10] proposed a blurred finger-vein image restoration method that considers both optical and scattering blur using PSF and CLS filters. They restored blurred finger-vein images by considering both optical blur components and scattering blur components and improved recognition performance. However, this method requires that parameters should be accurately predicted when measuring two PSFs to improve performance, causing extensive processing time. Choi et al. [11] proposed a finger-vein recognition method by restoring the optical blur included in the original finger-vein image based on modified conditional GAN. This method has the advantage that it can be applied to images acquired from various environments but has the disadvantage that it does not consider more complex motion blur that can occur during image acquisition.

As such, most of the previous studies did not focus on motion blur that can occur from the movement of fingers in finger-vein recognition and did not consider the image restoration associated with the motion blur. Therefore, we propose a new method of restoring a motion blurred finger-vein image using the modified DeblurGAN and recognizing the restored image using a deep CNN.

Point spread functions (PSFs) for skin scattering and optically blurred images are completely different from that for motion blurred images [2,3,4,5,6,7,8,9,10,11,27,28]. Therefore, the methods developed for skin scattering or optically blurred images cannot be used directly to solve the motion blurring issue. In the case of the handcrafted feature-based method of Table 1, the PSFs for skin scattering or optically blurred images should be replaced by the PSF for motion blurred images with optimal parameters of PSF. In case of deep feature-based method of Table 1, the CNN and GAN models for skin scattering or optically blurred images should be retrained with motion blurred images in addition to the modification of layers or filters of CNN and GAN models.

Table 1 presents a comparison of the advantages and disadvantages of the proposed method and the previous studies.

## 3. Proposed Method

### 3.1. Overview of the Proposed Method

**Figure 1:**
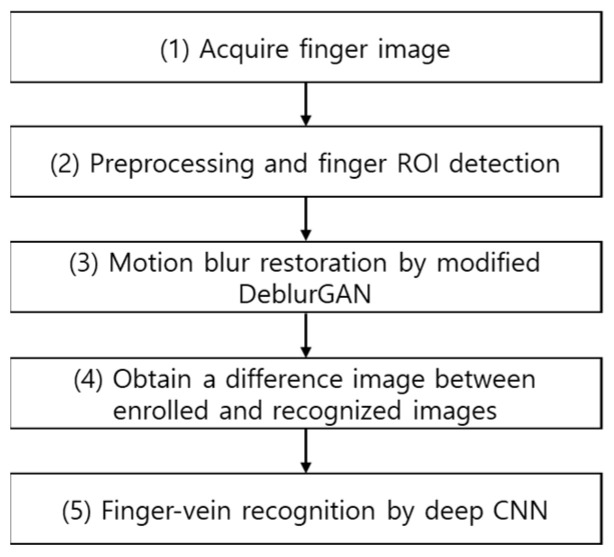
Flowchart of the proposed method.

Figure 1 shows the overall flowchart of the proposed method. After acquiring finger images (step (1)), the finger region of interest (ROI) is detected using preprocessing method (step (2)). Then, the motion blurred finger-vein image is restored using the proposed modified DeblurGAN (step (3)). One difference image is then generated from the restored enrolled and recognized images (step (4)). Lastly, based on the output score obtained by inputting the difference image in the deep CNN, finger-vein recognition is performed to distinguish genuine (authentic) or imposter matching (step (5)).

### 3.2. Preprocessing the Finger-Vein Image

**Figure 2:**
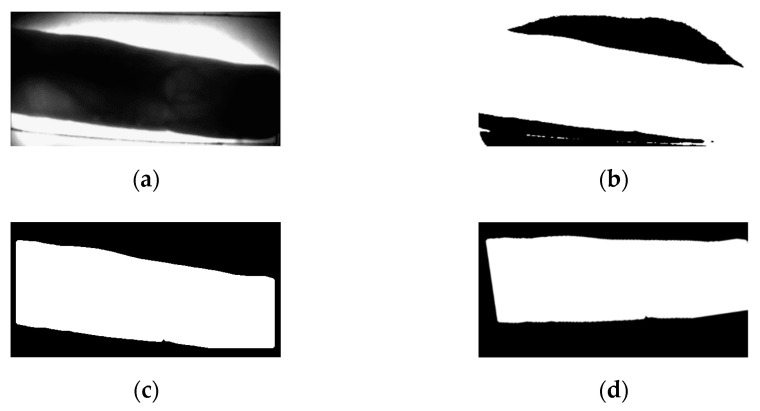
Example of background removal and in-plane rotation compensation: (**a**) original image; (**b**) binarized image; (**c**) background removed image; (**d**) in-plane rotation compensation.

The first part of preprocessing removes unnecessary background regions and finds the finger-vein ROI. The captured image is then binarized to obtain the image shown in Figure 2b. However, even if binarization is performed, the background is not completely removed, so an edge map is created using a Sobel filter. A difference image is then generated using the created edge map and the binarized image. By applying the area threshold method [29] to the generated difference image, an image with the background removed as shown in Figure 2c is obtained. Then, in order to correct misalignment caused by in-plane rotation of the finger image, which degrades recognition performance, second-order moments of the binarized mask *R* (Figure 2c), are calculated using Equation (1).

Note that f(x,y) and $\left(m_{x},\;m_{y}\right)$ represent image pixel values and central coordinates, respectively. Based on these values, the rotation angle *θ*, in Equation (2) is calculated to compensate for the in-plane rotation [30]. The compensated image, shown in Figure 2d, is obtained from this process.

(1) $$\begin{array}{c} a_{11}=\frac{\sum_{\left(x,y\right)\in R}{\left(y-m_{y}\right)}^{2}\cdot f\left(x,y\right)}{\sum_{\left(x,y\right)\in R}I\left(x,\;y\right)}\\ a_{12}=\frac{\sum_{\left(x,y\right)\in M}\left(x-m_{x}\right)\left(y-m_{y}\right)\cdot f\left(x,y\right)}{\sum_{\left(x,y\right)\in R}I\left(x,\;y\right)}\\ a_{22}=\frac{\sum_{\left(x,y\right)\in R}{\left(x-m_{x}\right)}^{2}\cdot f\left(x,y\right)}{\sum_{\left(x,y\right)\in R}I\left(x,\;y\right)} \end{array}$$

(2) $$\theta =\{\begin{array}{c} {tan}^{-1}\left\{\frac{a_{11}-a_{22}+\sqrt{{\left(a_{11}-a_{22}\right)}^{2}+4a_{12}^{2}}}{-2a_{12}}\right\}if\;a_{11}>a_{22}\\ {tan}^{-1}\left\{\frac{-2a_{12}}{a_{22}-a_{11}+\sqrt{{\left(a_{22}-a_{11}\right)}^{2}+4a_{12}^{2}}}\right\}if\;a_{11}\leq a_{22} \end{array}$$

**Figure 3:**
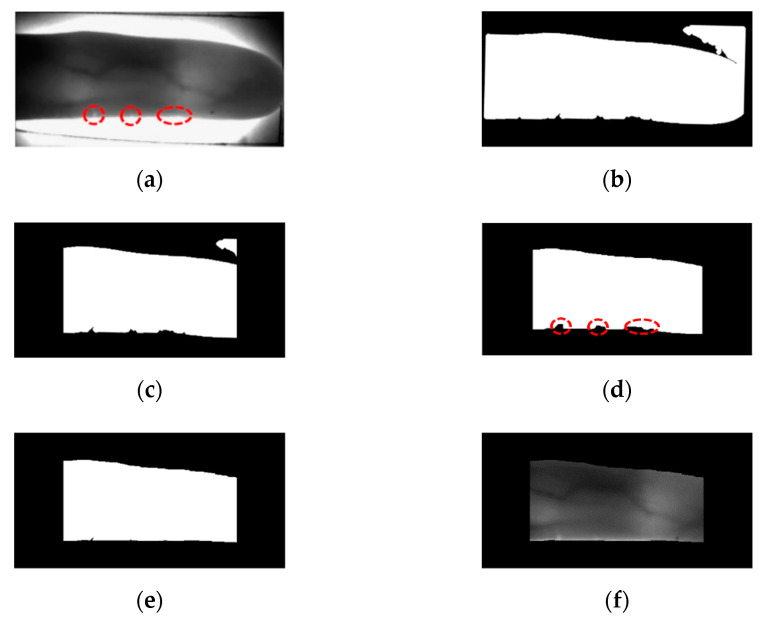
Extracting finger-vein ROI: (**a**) original finger image, (**b**) in-plane rotation compensation, (**c**) left and right areas removal, (**d**) component labeling, (**e**) ROI mask after filling black area of finger region, and (**f**) obtained ROI image.

As shown in Figure 3a, the left and right ends of the finger are the regions of the thick area or region with a fingernail where NIR lighting is not well-transmitted. Thus, these regions are inappropriate for recognition because vein patterns are not likely to be captured accurately. Therefore, the image, shown in Figure 3c, is obtained by removing the left and right sides by a predetermined size to which in-plane rotation compensation is applied. By performing erosion operation, component labeling process, and dilation operation [27], the unnecessary region for finger-vein recognition such as the upper right corner of Figure 3c, is removed. As a result of this process, an image as shown in Figure 3d is created. Since the vein pattern is not acquired by bright illumination, the black area of the finger area is not required for recognition. An ROI mask is obtained by using a 4 × 20 mask to fill the black area with the average pixel values around it (Figure 3e). In details, as shown in the red-dashed circles of the lower boundary of finger in Figure 3a, there exists bright pixels inside of finger caused by excessive illumination, which causes the error of binarization of lower boundary as shown in Figure 3b–d. Therefore, we applied 4 × 20 mask to the binarized image of Figure 3d. At each convolution position of mask, the average pixel value within 4 × 20 area (except for the black pixels of Figure 3d) is assigned to the binarized image of Figure 3d. That is, if the majority pixels within 4 × 20 area is white (255), white pixel is assigned. Then, the inaccurate black pixels of the red-dashed circles of Figure 3d are replaced by the white pixels of finger region as shown in the lower boundary of Figure 3e.

The mask size (4 × 20) generalizes on the images of different resolutions. To confirm this, we used two open databases of the Shandong University homologous multi-modal traits (SDUMLA-HMT) finger-vein database [31] and the Hong Kong Polytechnic University finger-image database version 1 [29] in our research.

### 3.3. Modified DeblurGAN-Based Finger-Vein Image Restoration

The principal objective of enhancement is to process the image so that the result is more suitable than the original image for a specific application [27]. Therefore, although image enhancement is mostly a subjective process, while image restoration is a generally objective process. Because image restoration is an attempt to reconstruct a degraded image using prior knowledge of degradation, the restoration method must focus on applying degradation modeling to restore the original image and the inverse process. The blur model based on the above process can be expressed as follows [28]:

(3) g(x, y)=h(x, y)∗f(x, y)+η(x, y)

Here, g(x, y) is a degraded (blurred) image, h(x, y) is a spatial representation of a degradation function (H), ∗ is a convolution operation, f(x, y) is an input image, and η(x, y) is an additive noise. If the above conditions are given, the goal of restoration is to obtain $\hat{f}\left(x,\;y\right)$, which is the estimation of an original image. The more accurately h(x, y) and η(x, y) are estimated, $\hat{f}\left(x,\;y\right)$ and f(x, y) become closer [28]. However, from g(x, y), which is the image obtained from various environments, it is extremely difficult to estimate h(x, y) and η(x, y) accurately. Furthermore, when images having different characteristics than those used for estimation are input, the estimated h(x, y) and η(x, y) may sometimes not be applicable. Considering these facts, this study proposes a training-based restoration model, the modified DeblurGAN, and we aim to ensure the restored finger-vein image $F^{res}$, becomes similar to the original finger-vein image $F^{ori}$, through training without separately estimating h(x, y) and η(x, y) when a motion blurred finger-vein image $G^{blur}$, is given.

**Figure 4:**
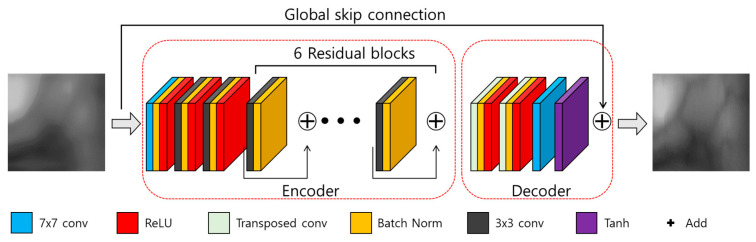
Generator of the modified DeblurGAN.

**Figure 5:**
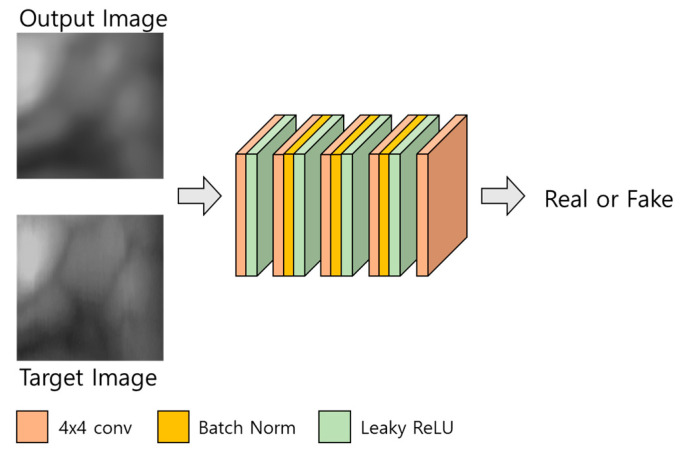
Discriminator of the modified DeblurGAN.

A deblurring task can be generally divided into blind and non-blind deblurring. For the non-blind deblurring method, deblurring is performed assuming that the blur kernel (h(x, y)) is known, whereas, for the blind deblurring method, deblurring is performed assuming that the blur kernel is not known [12]. In a general environment, a blind kernel is not known, and it is time-consuming to directly estimate it. In this study, we assume that the blur kernel is unknown, similar to the general environment. Also, it proposes a restoration method applicable for motion blurred finger-vein images obtained from various environments, so this study can be considered a blind deblurring task. Because the original DeblurGAN exhibits good performance in a blind motion-deblurring task [12], we determined that it would be effective in this study as well. Therefore, we propose a modified DeblurGAN. The generator of the modified DeblurGAN used in this study is shown in Figure 4 and Table 2, and the discriminator is shown in Figure 5 and Table 3. A more detailed explanation is provided in the next subsection.

#### 3.3.1. Generator

A GAN generally comprises generator and discriminator models in which the adversarial training between the two gradually improves the performance of both. The generator of the original DeblurGAN has one convolution block, two strided convolution blocks with strides of 1/2, nine residual blocks (ResBlocks) [32], and two transposed convolution blocks [12]. Each ResBlock consists of a convolution layer, an instance normalization layer, and a rectified linear unit (ReLU) for activation [33]. Compared with the original DeblurGAN, the following two aspects were modified for this study.

First, a dropout [34] is removed. In the original DeblurGAN, a dropout ratio of 0.5 is applied to each residual block of the generator, and the same ratio is applied for inference. Generally, a dropout is effective as a regularization method for avoiding overfitting, but it can cause the modification of a vein pattern in the restored output image, due to the randomness of a dropout when applied to a restoration task. The modified vein pattern then has different features from the original finger-vein image, which results in degraded performance. Rather than creating a variety of outputs in which the vein pattern is deformed, the generated pattern information needs a deterministic output that is similar to the original as possible, therefore, dropout.

**Figure 6:**
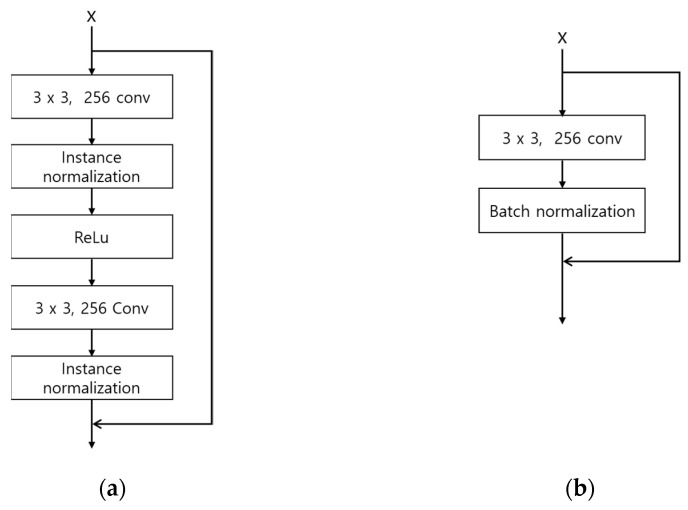
Architectures of original and modified residual blocks: (**a**) residual block in original DeblurGAN; (**b**) a modified residual block in the modified DeblurGAN.

Second, the number of parameters is reduced by modifying the residual blocks. Large parameters can increase the inference time when applied to an actual environment, and increased inference time can cause the inefficiency of the system. The original DeblurGAN used the GoPro [35] and Kohler datasets [36] and applied nine residual blocks to the generator. In this study, the existing nine residual blocks were reduced to six to shorten the inference time by reducing the number of parameters. Also, by modifying the residual blocks as shown in Figure 6, feature information is maintained in the layer prior to the next convolution layer, and the number of parameters is reduced. The width and height of feature map are reduced by passing through convolution layer, which usually causes the reduction of important feature information [32]. Therefore, by comparing Figure 6a,b, the second 3 × 3, 256 Conv layer is removed in our modified residual block, which can maintain feature information in the layer prior to the next convolution layer. In addition, the number of parameters is reduced by removing the second 3 × 3, 256 Conv layer in the modified residual block. Consequently, the number of parameters of the generator is reduced from 6.0 to 4.2 million.

#### 3.3.2. Discriminator

The structure of the discriminator is shown in Figure 5 and Table 3. The discriminator of the modified DeblurGAN proposed in this study has the same structure as the discriminator of the original DeblurGAN, which used the Wasserstein WGAN gradient penalty (GP) [37]. For a GAN, the Nash equilibrium in a non-convex system must be found using continuous and high-dimensional parameters for smooth training, however, the existing GAN [38] cannot solve this problem, therefore, it fails to converge [39]. In the case of DeblurGAN, WGAN-GP is used as a critic function using Wasserstein distance and the gradient penalty methods proposed in [37]. Thus, a structure that is robust to generator structure selection and at the same time enables stable training is proposed. In this study, these advantages of the discriminator of the original DeblurGAN are adopted.

#### 3.3.3. Loss

In the case of the original DeblurGAN, a perceptual loss is applied to perceptually hard to distinguish between the generated image and the real sharp image and to restore finer texture detail [12]. A perceptual loss refers to the difference in feature maps between the generated and target images, which can produce better results than the loss that generates blurry results by calculating the pixel-wise average difference such as L1 or L2 loss. The perceptual loss function used in this study, based on the previous study [12], can be defined as follows:

(4) $$L_{X}=\frac{1}{W_{\emptyset }H_{\emptyset }}\overset{W_{\emptyset }}{\underset{x=1}{\sum }}\overset{H_{\emptyset }}{\underset{y=1}{\sum }}{\left(\emptyset {\left(F^{ori}\right)}_{x,y}-\emptyset {\left(G_{\theta_{G}}\left(F^{blur}\right)\right)}_{x,y}\right)}^{2}$$

where ∅ is the feature maps extracted from the ImageNet pretrained network. For the original DeblurGAN, the feature maps extracted from the third convolution layer before the third max-pooling layer in the visual geometry group (VGG)-19 [40] are used for perceptual loss. $W_{\emptyset }$ and $H_{\emptyset }$ are the width and height of feature maps, respectively. In a classification network, such as that of a VGG, abstracted features extracted from a higher layer preserve the overall spatial structure, whereas low-level features, such as color, corner, edge, and texture, cannot be preserved [41,42]. In terms of finger-vein images, it is important to restore the high-level features of restored output image similar to those of the original image, however, restoring low-level features is important as well, because vein patterns and texture are slightly different for each class, and performance can be varied due to differences in low-level features during recognition. Because of these reasons, unlike the original DeblurGAN that applied perceptual loss by extracting feature maps from the middle layer of the ImageNet pretrained VGG-19, in this study, feature sets are extracted from the generated image and target image in the first residual block (conv2_x) using the ImageNet pretrained ResNet-34 [32] model, respectively, and the difference between the two feature sets is applied as a perceptual loss. In a typical neural network, vanishing gradient and explosion occur as the layer gets deeper, eventually resulting in performance degradation. In ResNet, however, this problem is solved by applying a residual learning method. The residual block applying the residual learning method is trained so that identity mapping F(x)+x that is mapping between output F(x) of the weight layer and output x of the layer just before the weight layer, and plain layer output H(x) are the same (H(x)=F(x)+x). From the characteristics of the residual block that identity mapping the output information of the previous layer to the next layer, we inferred that low-level features such as color, corner, edge, and texture of the finger-vein can be preserved during restoration training. For this reason, a perceptual loss is applied from the conv2_x layer of ResNet-34 instead of the original VGG-19.

#### 3.3.4. Summarized Differences between Original DeblurGAN and Proposed Modified DeblurGAN

The differences between the original DeblurGAN and the proposed modified DeblurGAN are as follows.

- A dropout is applied to the generator of the original DeblurGAN, whereas a dropout is not applied to the generator of the modified DeblurGAN because the vein patterns of the restored image can be modified. The dropout layer usually helps avoiding overfitting. However, the dropout layer can also bring about the excessive sparsity of activation and features with coarser features compared to the case without the dropout layer [34,43], which can cause the consequent modification of a vein pattern in the restored output image. Therefore, we do not use the dropout layer in the generator of proposed modified DeblurGAN.
- In the original DeblurGAN, nine residual blocks (convolutional layer—normalization layer—activation layer—convolutional layer—normalization layer) were used for the generator. In the modified DeblurGAN, to reduce the inference time, the number of parameters was reduced by reducing the structure of the residual block (convolutional layer-normalization layer) and reducing the total number of residual blocks to six.
- In the original DeblurGAN, high-level feature maps extracted from the third convolution layer prior to the third max-pooling layer of the ImageNet-pretrained VGG-19 were applied to a perceptual loss. However, it is equally important to restore the information of low-level features, such as color, corner, edge, and texture, during finger-vein restoration. Hence, a perceptual loss was applied to the first residual block (conv2_x) using the ImageNet-pretrained ResNet-34 in the modified DeblurGAN.

### 3.4. Finger-Vein Recognition by Deep CNN

**Figure 7:**
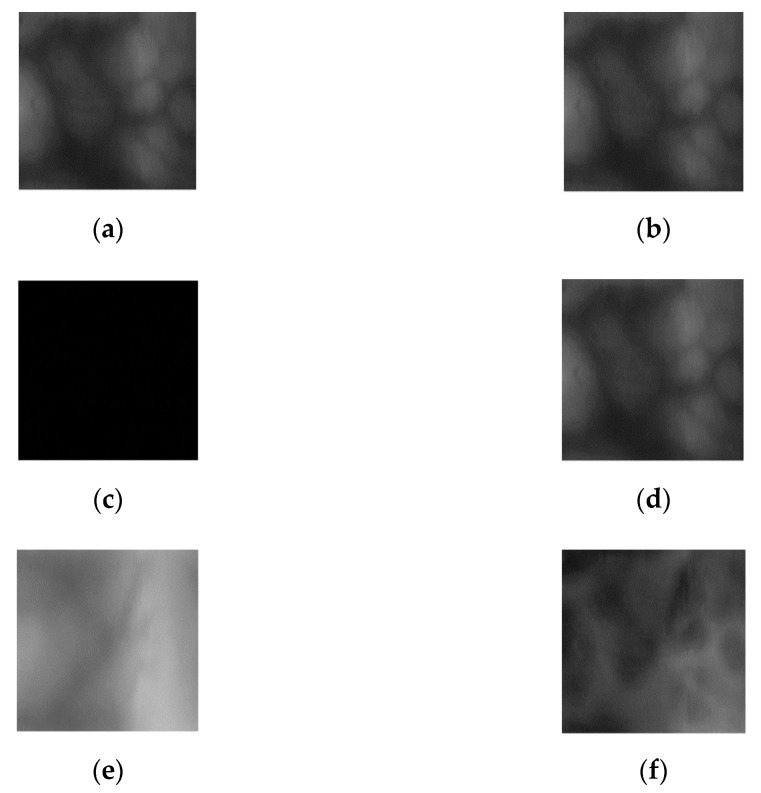
Difference images of registered and input images. (**a**) Registered image, (**b**) input image of same class as registered image, (**c**) difference image of (**a**,**b**), (**d**) registered image, (**e**) input image of different class as registered image, and (**f**) difference image of (**d**,**e**).

In this study, the difference image between registered (enrolled) and recognized images was used as an input for CNN-based finger-vein recognition. An image differencing method determines the changes in images where the differences are determined by calculating the pixel differences, and a new image is then created based on the calculation results [44]. Thus, an image differencing method reacts sensitively to the changes in images. For the finger-vein datasets used in this study, if the same class images are used, the pixel difference between the two images is small. So, in general, a pixel value with a low difference image, that is, an image with many black areas is an output. Whereas in the case of other classes, since the pixel difference between the two images is large, the difference image has generally a high pixel value, that is, an image with many bright areas is output. An image differencing method has the advantage of expressing the characteristics of genuine and imposter matching with one output image. Here, genuine matching refers to matching when the input image and the enrolled image are the same class, and imposter matching refers to matching when the input image and the enrolled image are the different class. The finger-vein datasets used in this study have a high similarity of vein patterns between intra-class, but a low similarity between inter-class. Therefore, the finger-vein recognition performance can be verified in the difference image. The examples of finger-vein difference images generated from the dataset used in this study are shown in Figure 7c,f.

The generated difference image is then used as an input to deep CNN. DenseNet-161 [45] is used to recognition of finger-vein images. DenseNet adopts dense connectivity in which the feature maps of a previous layer are concatenated in the current layer.

(5) $$x_{l}=H_{l}\left(\left[x_{0},x_{1},\ldots ,x_{l-1}\right]\right)$$

Equation (5) represents dense connectivity, where $\left[x_{0},x_{1},\ldots ,x_{l-1}\right]$ means the feature map concatenation from layers 0 to *l* − 1. A dense block performs feature map concatenation of the previous and the current layer and transfers the concatenated feature maps to the following layer. $H_{l}$ is a composite function and is composed of batch normalization [46], ReLU [33], and a convolution layer. Generally, as the network becomes deeper, the number of channels of feature maps caused by dense connectivity increases, resulting in an increased number of network parameters. To mitigate the increasing parameters, a bottleneck layer is added to the dense block of DenseNet. As a result, utilizing the bottleneck structure reduces computational costs. However, the output of a dense block concatenates all layers within the block. As the layer gets deeper or the number of layers in the dense block increases, the size and depths of the feature map increase enormously. To solve this problem, a transition layer was added between the dense blocks to reduce the size and depths of the feature maps. The transition layer cuts the number of feature map depths by half through 1 × 1 convolutional computation and reduces width and height by half using 2 × 2 average pooling. In addition, by specifying a growth rate, DenseNet controls the number of output feature map channels. Dense block outputs the feature map at the size of the designated growth rate. In this research, the growth rate is set to 48.

In this study, for finger-vein recognition, the DenseNet-161 pretrained with the ImageNet database [47] is fine-tuned with the finger-vein training data. Difference images are used for the training and testing process, and these images are created using the output restored images by the proposed modified DeblurGAN. The number of output classes of DenseNet-161 is set to 2, genuine matching and imposter matching. The criterion for this is based on the output score obtained from the last layer of the DenseNet. With respect to the threshold of the equal error rate (EER) of genuine and imposter matching distributions of the CNN output score obtained from the training data, it is determined as genuine matching if the CNN output score of the testing data is below the threshold. And imposter matching is determined if the output score is greater than the threshold. The EER is the rate of error at the point where the false rejection rate (FRR) which is the error rate of falsely rejecting genuine matching as an imposter matching and the false acceptance rate (FAR) which is the error rate of falsely accepting imposter matching as genuine matching are equal.

## 4. Experimental Results

### 4.1. Two Open Databases for Experiments

**Figure 8:**
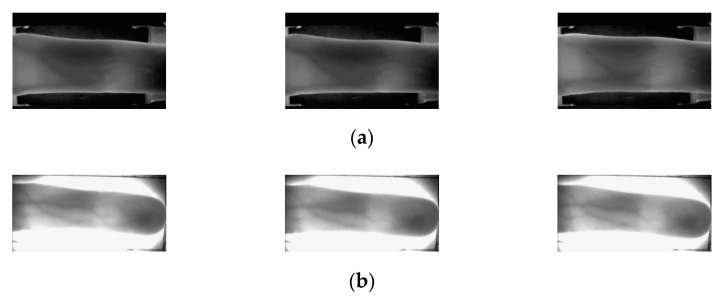
Images obtained from the same finger. (**a**) SDU-DB and (**b**) PolyU-DB.

In this study, experiments were conducted using two types of open finger-vein databases, SDUMLA-HMT finger-vein database [31] and session 1 images from the Hong Kong Polytechnic University finger-image database version 1 [29]. In SDUMLA-HMT finger-vein database, 6 images from the ring, middle, and index finger from both hands were obtained respectively, from 106 individuals, a total of 3816 images were obtained (2 hands × 3 fingers × 6 images from 106 individuals). In session 1 from the Hong Kong Polytechnic University finger-image database version 1, 6 images from the middle and index finger images were obtained respectively, from 156 individuals, a total of 1872 images were obtained (2 fingers × 6 images from 156 individuals). In this study, the finger-vein database of the SDUMLA-HMT is referred to as SDU-DB, and the session 1 finger-image database version 1 of the Hong Kong Polytechnic University is referred to as PolyU-DB. Figure 8 shows examples from the same finger for PolyU-DB and SDU-DB. The image resolution of SDUMLA-HMT is 320 × 240 pixels, and that of the Hong Kong Polytechnic University finger-image database is 513 × 256 pixels.

SDU-DB consists of 636 classes, whereas PolyU-DB consists of 312 classes. All experiments adopted two-fold cross-validation. Through the two-fold cross-validation method, data of the same class were not used for training and testing (open-world setting). The average accuracy measured through two-fold cross-validation was adopted as the final recognition accuracy.

### 4.2. Motion Blur Datasets for Finger-Vein Image Restoration

**Figure 9:**
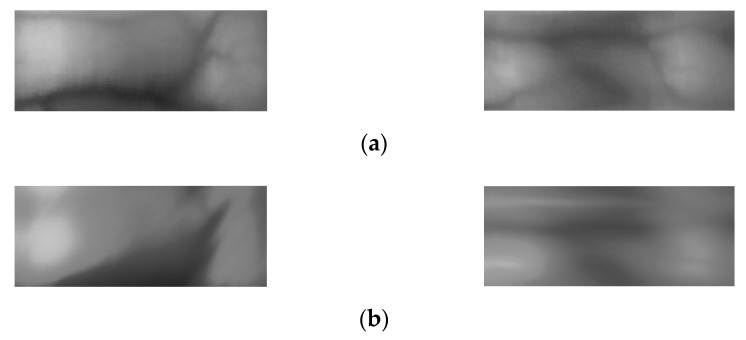
Examples of original images and motion blurred images of SDU-DB. (**a**) Original images; (**b**) motion blurred images.

**Figure 10:**
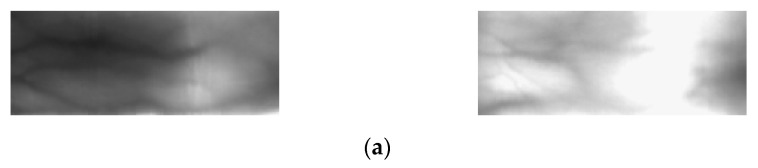

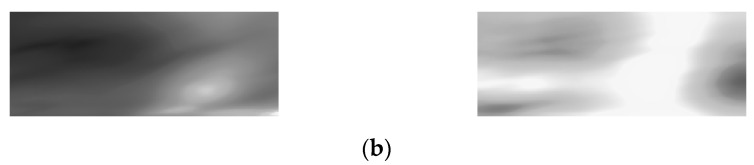
Examples of original images and motion blurred images of PolyU-DB. (**a**) Original images; (**b**) motion blurred images.

In the case of PolyU-DB and SDU-DB, which are open databases used in this study, motion blurred finger-vein datasets were not constructed. Therefore, to proceed with this study, a motion blurred finger-vein database was constructed by applying motion blurring kernels to the two open databases. When constructing the database, non-uniform (random) motion blurring kernels were applied instead of uniform motion blurring kernels to closely resemble the actual environment. For the random motion blurring kernels, the method proposed by Kupyn et al. [12] was used. Figure 9 and Figure 10 show original and generated motion blurred images of SDU-DB and PolyU-DB.

### 4.3. Data Augmentation and Experimental Setup

The datasets used in this study do not contain enough images to train a deep CNN, which would result in overfitting. To solve this problem, a data augmentation method was applied to increase the number of training data. For this method, 5 pixel shifting was applied for each image based on 8 directions in a combination of the top, bottom, left, and right. Therefore, each image was increased to 9 times including the original image. Table 4 presents the descriptions of original and augmented data from PolyU-DB and SDU-DB datasets. From the data augmentation, 54 images were generated that increased 9 times from 6 images per class. When training DenseNet-161 for finger-vein recognition, only 1 image among 54 augmented images was selected as an enrolled image, and the other images were used as input images. A difference image was generated using the enrolled image and input image to determine genuine and imposter matching. In the case of SDU-DB, the number of imposter matching was 317 times that of genuine matching, and it was 155 times that of genuine matching for the PolyU-DB. When training this data as it is, a bias on the majority class occurs due to data imbalance. In order to solve this problem, when genuine matching data is augmented with the same number as imposter matching data, training time is increased due to a large number of data, and an overfitting problem for genuine matching data can occur. Therefore, in this study, we applied a random selection method for the imposter matching data. Augmentation and random selection methods were applied to both SDU-DB and PolyU-DB in the same manner, but only to the training data. The original images that were not augmented were used as the testing data.

The training and testing were performed on a desktop computer equipped with NVIDIA GeForce GTX 1070 graphics processing unit (GPU) [48] and Intel^®^ Core™ i7-9700F CPU with 16 GB RAM.

### 4.4. Training of Modified DeblurGAN Model for Motion Blur Restoration

**Figure 11:**
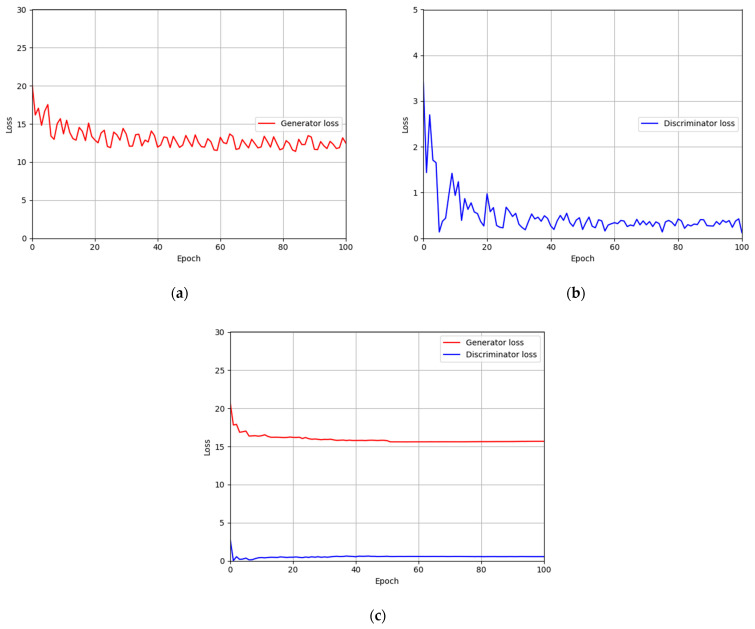
Training and validation loss graphs of the modified DeblurGAN (SDU-DB): training loss graphs of (**a**) generator and (**b**) discriminator. (**c**) Validation loss graphs of generator and discriminator.

**Figure 12:**
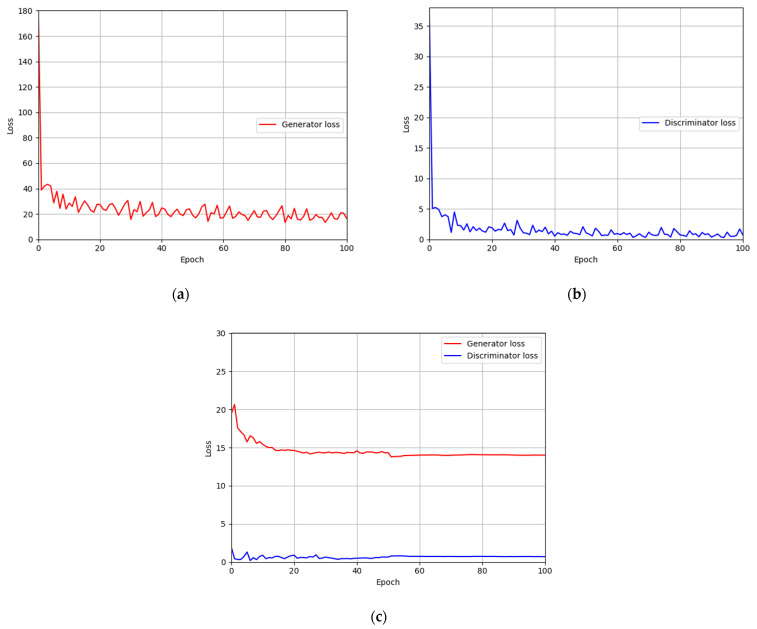
Training and validation loss graphs of the modified DeblurGAN (PolyU-DB): training loss graphs of (**a**) generator and (**b**) discriminator. (**c**) Validation loss graphs of generator and discriminator.

For the training parameter of modified DeblurGAN, the max epoch was set to 100, the mini-batch size was set to 4, and the learning rate was set to 0.0005. Adaptive moment estimation (Adam) optimization [49] was used for the generator and discriminator to train the modified DeblurGAN. Figure 11a,b and Figure 12a,b show the graphs of training loss of the proposed modified DeblurGAN according to the epoch for SDU-DB and PolyU-DB, respectively. The loss values converged as the training progresses, confirming that the proposed modified DeblurGAN was trained sufficiently, as shown in the figures. The trained model with excessive larger number of epochs usually causes the model overfitting. Therefore, we used 10% of training data as validation set which was not used as training. With the trained model of each epoch, the accuracies of validation set was measured, and the model which showed the best validation accuracy was selected for measuring testing accuracy with testing data. We included the validation performances with validation set in Figure 11c and Figure 12c, which confirms that our model was not overfitted with training data.

### 4.5. Training of DenseNet-161 for Finger-Vein Recognition

A stochastic gradient descent (SGD) optimization method [50] was used to train the CNN model for finger-vein recognition. This method involves multiplying a gamma value by the learning rate for every step size at a mini-batch unit to reduce the learning rate, thereby rapidly converging training accuracy and loss. As explained in Section 3.4, DenseNet-161 was used in this study for training and testing. The number of output classes was set to two (authentic and imposter-matching), the number of max epochs was set to 30. The mini-batch size was set to 4, the learning rate was set to 0.001, the step size was set to 16 epochs, the momentum was set to 0.9, and the gamma value was set to 0.1. All the hyperparameters were determined with training data. In detail, the optimal hyperparameters (with which the highest accuracies of finger-vein recognition were obtained with training data) were selected. The search spaces for the number of max epochs, mini-batch size, and learning rate were 10~50, 1~10, and 0.0001~0.01, respectively. The search spaces for the step size, momentum, and gamma value are 5~25 epochs, 0.1~1, and 0.1~1, respectively.

**Figure 13:**
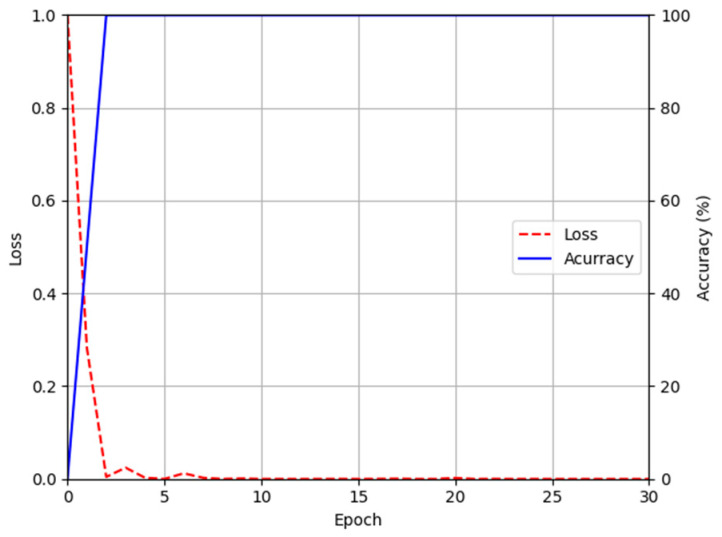
Training accuracy and loss graphs of DenseNet-161 using images restored by proposed modified DeblurGAN (SDU-DB).

**Figure 14:**
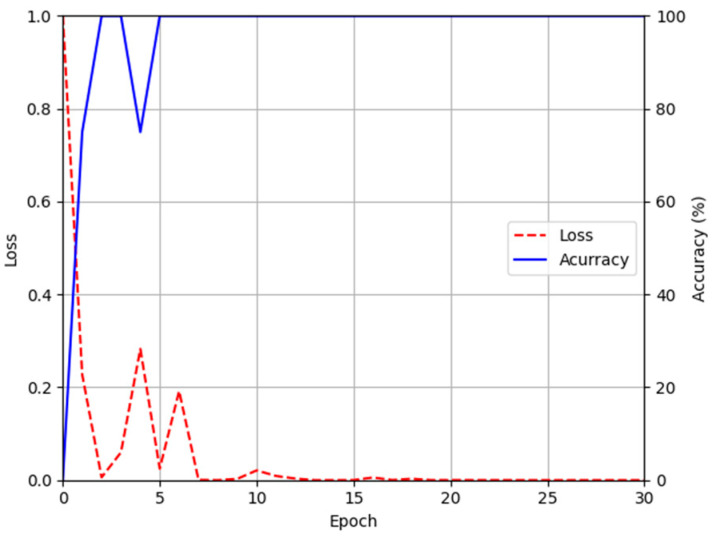
Training accuracy and loss graphs of DenseNet-161 using images restored by proposed modified DeblurGAN (PolyU-DB).

Figure 13 and Figure 14 show the training loss and accuracy graphs of DenseNet-161, which used a difference image restored by the modified DeblurGAN as input. As shown in the training graphs, training loss converged to nearly zero, whereas accuracy converged to nearly 100, indicating that the CNN model for finger-vein recognition was sufficiently trained.

### 4.6. Testing Results of Proposed Method

#### 4.6.1. Ablation Studies

As ablation studies, experiments were conducted according to with or without motion blur is applied, and the methods can be largely divided into 4 schemes. Scheme 1 means that DenseNet-161 trained with the original training data without blurring was used to perform finger-vein recognition with the original testing data to measure the EER. Scheme 2 means that DenseNet-161 trained with the original training data was used to perform finger-vein recognition with the motion blurred testing data to measure the EER. Scheme 3 represents that DenseNet-161 trained with the motion blurred training data was used to perform finger-vein recognition with the motion blurred testing data to measure the EER. Lastly, scheme 4 represents that DenseNet-161 trained with the training data restored with the modified DeblurGAN proposed in this study was used to perform finger-vein recognition with testing data restored using the modified DeblurGAN to measure the EER. As shown in schemes 2 and 3 in Table 5 and Table 6, the vein-pattern region and other regions were difficult to distinguish, due to motion blur, resulting in degradation of recognition performance. Also, in all cases, compared with schemes 2 and 3, when training was performed with the training data restored with the modified DeblurGAN, and recognition was performed for the testing data restored with the modified DeblurGAN, the recognition accuracy was the highest in scheme 4.

**Figure 15:**
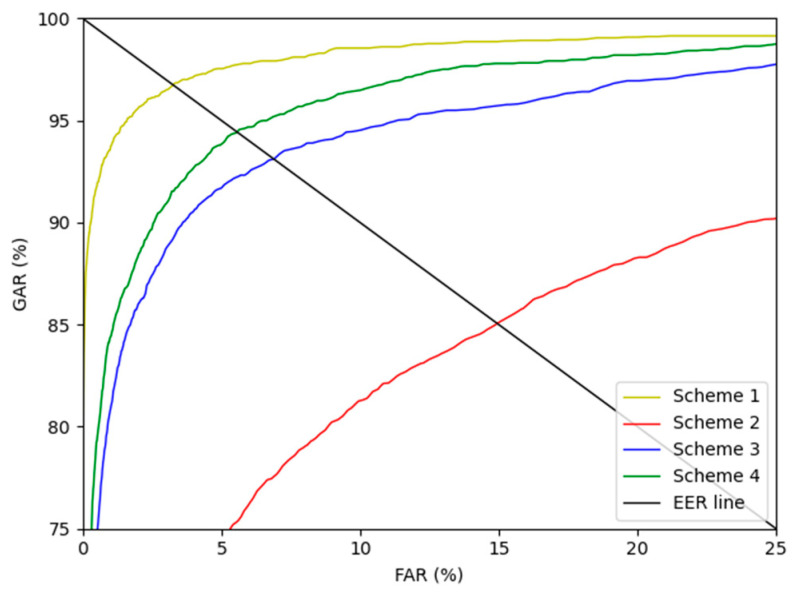
SDU-DB finger-vein recognition ROC curve for scheme 1–4.

**Figure 16:**
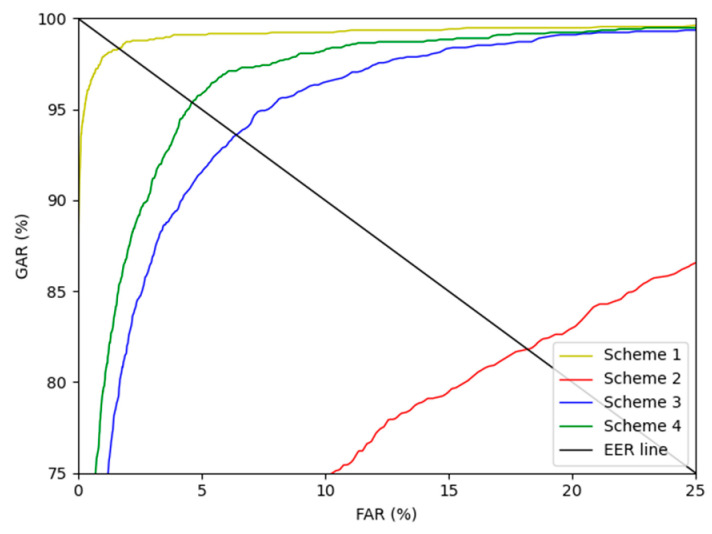
PolyU-DB finger-vein recognition ROC curve for schemes 1–4.

Figure 15 and Figure 16 show the receiver operating characteristics (ROC) curves for the recognition performance of schemes 1–4 of SDU-DB and PolyU-DB, respectively. Here, GAR is calculated as 100—FRR (%). As shown in Figure 15 and Figure 16, in all cases, the recognition performance after restoration with the modified DeblurGAN proposed in this study (scheme 4) was higher than schemes 2 and 3.

In Table 7 and Table 8, the recognition performances of the modified DeblurGAN model were compared according to the changes in the perceptual loss based on the features extracted from the various CNN models and layers. For a fair performance evaluation, the same recognition model was used for all cases based on scheme 4 to measure the recognition accuracy. For VGG-19 (original DeblurGAN), features extracted from the third convolution layer before the third max-pooling were used. Moreover, features extracted from the first convolution layer before the third max-pooling were used for VGG-19 (conv3.1). This is a result of reflecting the features extracted from a layer prior to VGG-19 (original DeblurGAN) in the perceptual loss, indicating that VGG-19 (original DeblurGAN) showed better recognition performance. For ResNeXt-101 (conv2), better recognition performance was exhibited over VGG-19 (original DeblurGAN) and VGG-19 (conv3.1) for both experiments. Lastly, for ResNet-34 (conv2_x), the features extracted from the first residual block (conv2_x) were applied to a perceptual loss (proposed method), thus exhibiting the best performance in all cases with SDU-DB whereas VGG-19 (conv3.1) shows the better accuracies than other cases with PolyU-DB.

#### 4.6.2. Comparisons with the State-of-the-Art Methods

For the next experiment, the similarities between the images restored with the state-of-the-art methods and the proposed modified DeblurGAN and the original images were quantitatively evaluated. For a numerical comparison, a signal-to-noise ratio (SNR) [52], peak SNR (PSNR) [53], and SSIM [54] were measured. SNR and PSNR are evaluation metrics based on the MSE between two images. Equations (6)–(8) are mathematical equations of MSE, SNR, and PSNR, respectively.

(6) $$\mathrm{MSE}=\frac{1}{hw}\overset{h-1}{\underset{i=0}{\sum }}\overset{w-1}{\underset{j=0}{\sum }}{\left[I_{o}\left(i,\;j\right)-I_{r}\left(i,\;j\right)\right]}^{2}$$

(7) $$\mathrm{SNR}=10{log}_{10}\left(\frac{\frac{\sum_{i=0}^{h-1}\sum_{j=0}^{w-1}{\left[I_{o}\left(i,\;j\right)\right]}^{2}}{hw}}{MSE}\right)$$

(8) $$\mathrm{PSNR}=10{log}_{10}\left(\frac{255^{2}}{MSE}\right)$$

where *I_r_* is the restored image obtained from the state-of-the-art or proposed methods, and *I_o_* is the original image. *h* and *w* are the height and width of an image, respectively. Equation (9) is the mathematical equation of SSIM:

(9) $$\mathrm{SSIM}=\frac{\left(2\mu_{o}\mu_{r}+C_{1}\right)\left(2\sigma_{or}+C_{2}\right)}{\left(\mu_{o}{}^{2}+\mu_{r}{}^{2}+C_{1}\right)\left(\sigma_{o}{}^{2}+\sigma_{r}{}^{2}+C_{2}\right)}$$

where *μ_r_* and *σ_r_* are the mean and standard deviation of the pixel values of the restored image, respectively. *μ_o_* and *σ_o_* are the mean and standard deviation of the pixel values of the original image, respectively. *σ_or_* is the covariance of two images, and $C_{1}$ and $C_{2}$ are constants to prevent the denominator of each equation from becoming zero. Using the evaluation metrics of Equations (6)–(9), the enhancement quality of our proposed method and that of the state-of-the-art was numerically evaluated as shown in Table 9 and Table 10. As shown in Table 9 and Table 10, SRN-DeblurNet shows the higher values for PSNR, SNR, and SSIM compared to our modified DeblurGAN. That is, the qualities of restored images by SRN-DeblurNet are more similar to those of original ones than those by our method. However, the recognition accuracies by our method are higher than those by SRN-DeblurNet as shown in Table 11 and Table 12. That is because the additional noises are included in the restored image and the features similar to the original features cannot be restored by SRN-DeblurNet, which causes the degradation of recognition accuracies although the qualities of restored images are similar to those of original ones.

**Figure 17:**
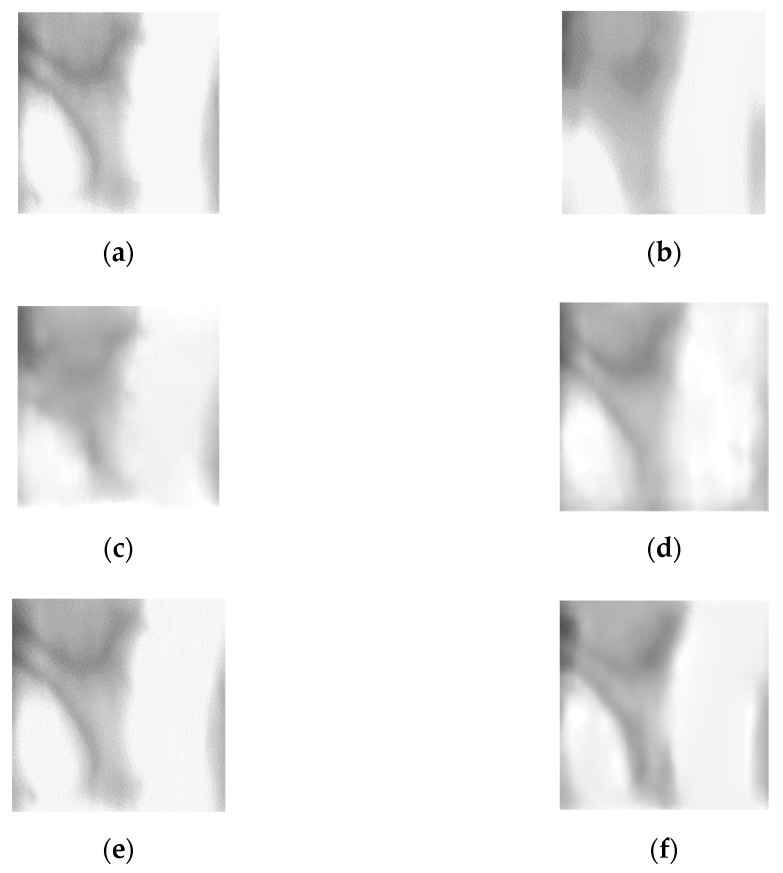
Examples of restored images using the state-of-the-art methods and the proposed modified DeblurGAN: (**a**) original images, (**b**) motion blurred images, and the restored images by (**c**) original DeblurGAN, (**d**) DeblurGANv2, (**e**) SRN-DeblurNet, and (**f**) proposed modified DeblurGAN.

Figure 17 shows examples of the finger-vein images restored by state-of-the-art methods and the modified DeblurGAN. For the next experiment, finger-vein recognition performances were compared using the images restored by the modified DeblurGAN and those restored by the state-of-the-art restoration methods for SDU-DB and PolyU-DB, as shown in Table 11 and Table 12. For the comparative experiment, the same recognition model was used for a fair performance evaluation to measure the recognition accuracy using the scheme 4 method of Table 5 and Table 6. As shown in Table 11 and Table 12, finger-vein recognition performance was higher than the existing state-of-the-art restoration methods, when the restoration was performed using the modified DeblurGAN method.

**Figure 18:**
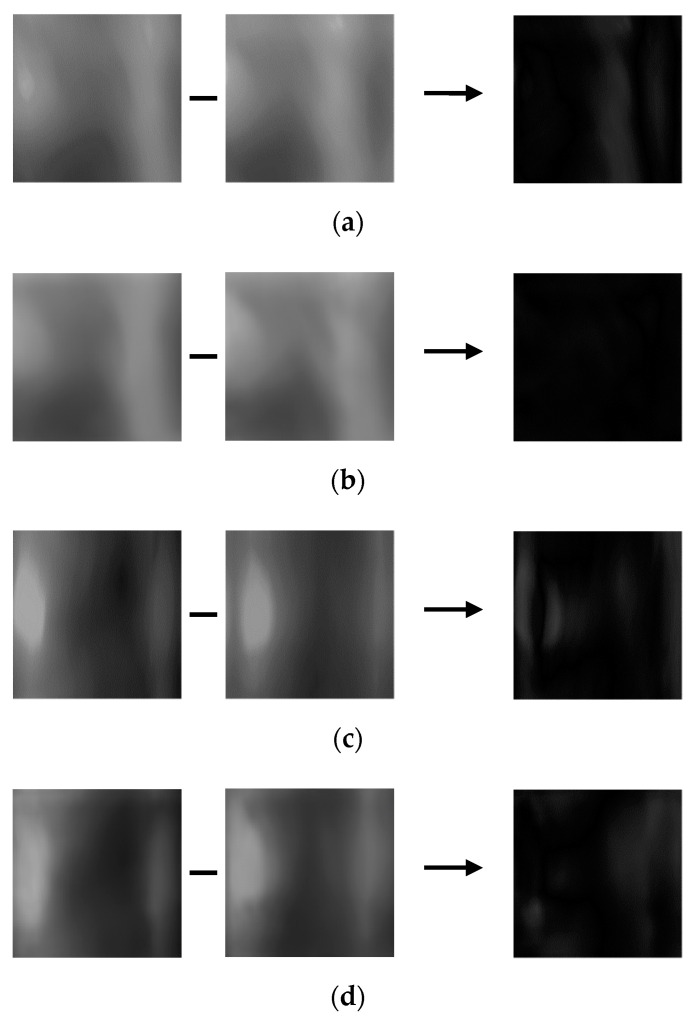
Correct recognition examples after restoring motion blur. (**a**) Incorrect genuine matching before restoring motion blur, (**b**) correct genuine matching after restoring motion blur, (**c**) incorrect imposter matching before restoring motion blur, and (**d**) correct imposter matching after restoring motion blur. From the left, examples in (**a**–**d**) present the registered, input, and difference images, respectively.

Figure 18a,c are the result of authentic and imposter matching prior to restoration, which provide incorrect matching results caused by modified vein patterns and texture information due to motion blur. Authentic matching was falsely rejected as imposter matching, whereas imposter matching was falsely accepted as authentic, thus decreased the recognition performance. Figure 18b,d are the results of correct matching by restoring the incorrect matching problem in (a) and (c) by the modified DeblurGAN. Authentic matching was classified as correct acceptance, and imposer matching was classified as correct rejection.

**Figure 19:**
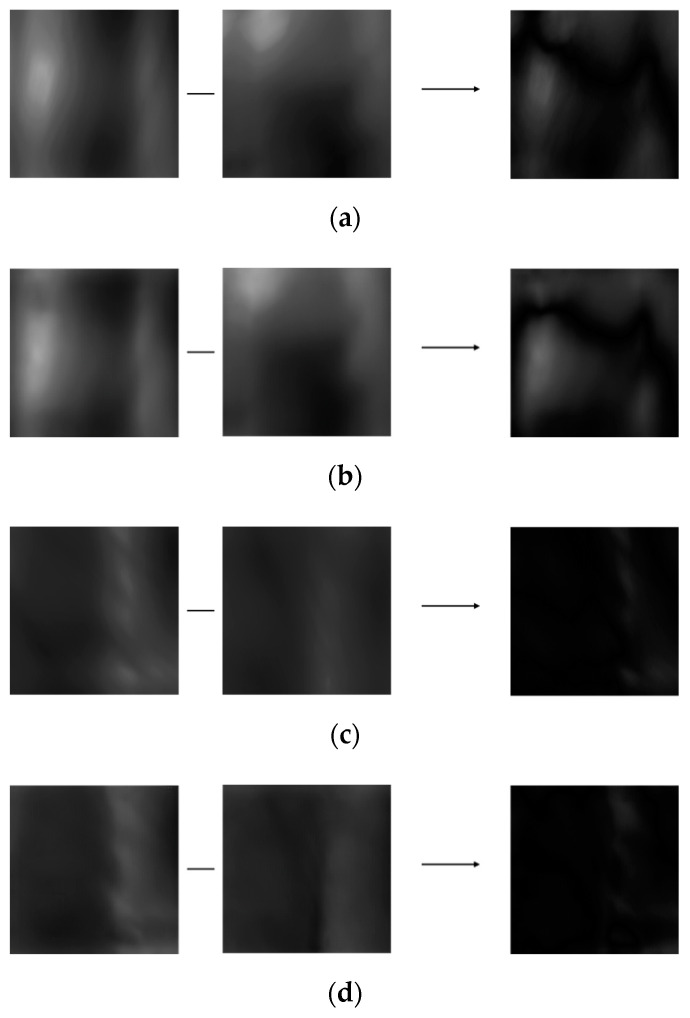
Incorrect recognition examples after restoring motion blur. (**a**) Incorrect genuine matching before restoring motion blur, (**b**) incorrect genuine matching after restoring motion blur, (**c**) incorrect imposter matching before restoring motion blur, and (**d**) incorrect imposter matching after restoring motion blur. From the left, examples in (**a**–**d**) present the registered, input, and difference images, respectively.

Figure 19 is an example of incorrect authentic matching and incorrect imposter matching despite the restoration method proposed in this study is applied. In the case of incorrect authentic matching, the difference in motion blur between the same classes is so severe that it is recognized as an imposter even after restoration, resulting in incorrect matching. In the case of incorrect imposter matching, the enrolled image and the input image appear similarly in dark shades, and the vein pattern is not clearly visible, so it recognized as authentic even after restoration, resulting in incorrect matching.

### 4.7. Processing Time of Proposed Method

**Figure 20:**
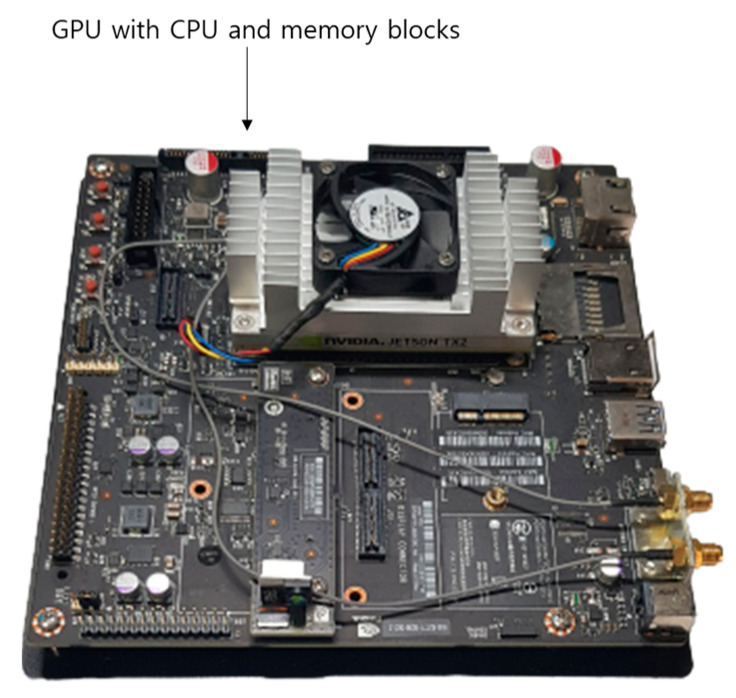
Jetson TX2 embedded system.

For the next experiment, the inference time of the modified DeblurGAN proposed in this study and DenseNet-161 for the finger-vein recognition method was measured. The measurements were taken on the desktop described explained in Section 4.3 and the Jetson TX2 embedded system [55] shown in Figure 20. The reason for measuring using the embedded system is that on-board edge computing, which operates as an embedded system attached to the entrance door, is involved for most access-controlled type finger-vein recognition systems. Thus, it must be verified that on-board computing is feasible on the system proposed. Jetson TX2 has an NVIDIA Pascal^TM^-family GPU (256 CUDA cores), with 8-GB memory shared between the CPU and GPU, and 59.7-GB/s of memory bandwidth. It uses less than 7.5 watts of power. As presented in Table 13, in the case of the method proposed in this study, the recognition speed for one image was 16.2 ms on a desktop computer and 232.3 ms on the Jetson TX2 embedded system. This corresponds to 61.72 frames/s (1000/16.2) and 4.3 frames/s (1000/232.3), respectively. The processing time on the Jetson TX2 embedded system was longer than the desktop computer, due to limited computing resources. However, through the experiment, it was confirmed that the proposed method is applicable to an embedded system having limited computing resources.

### 4.8. Analysis of Feature Map

#### 4.8.1. Class Activation Map of Restored Image

**Figure 21:**
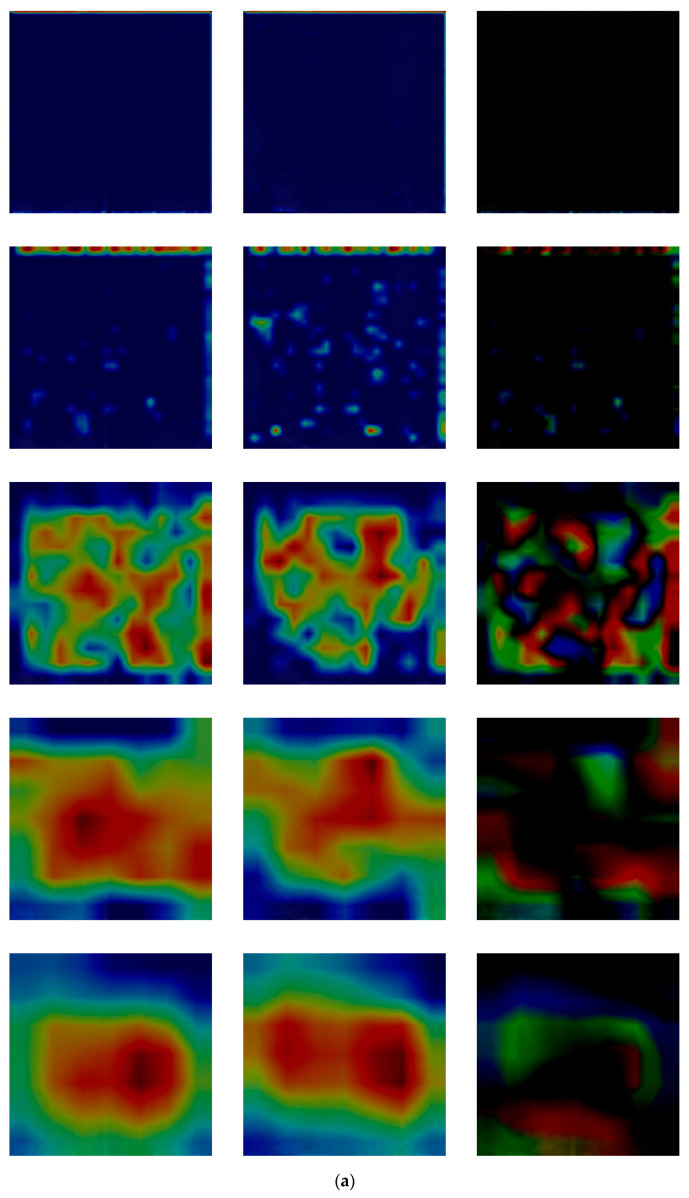

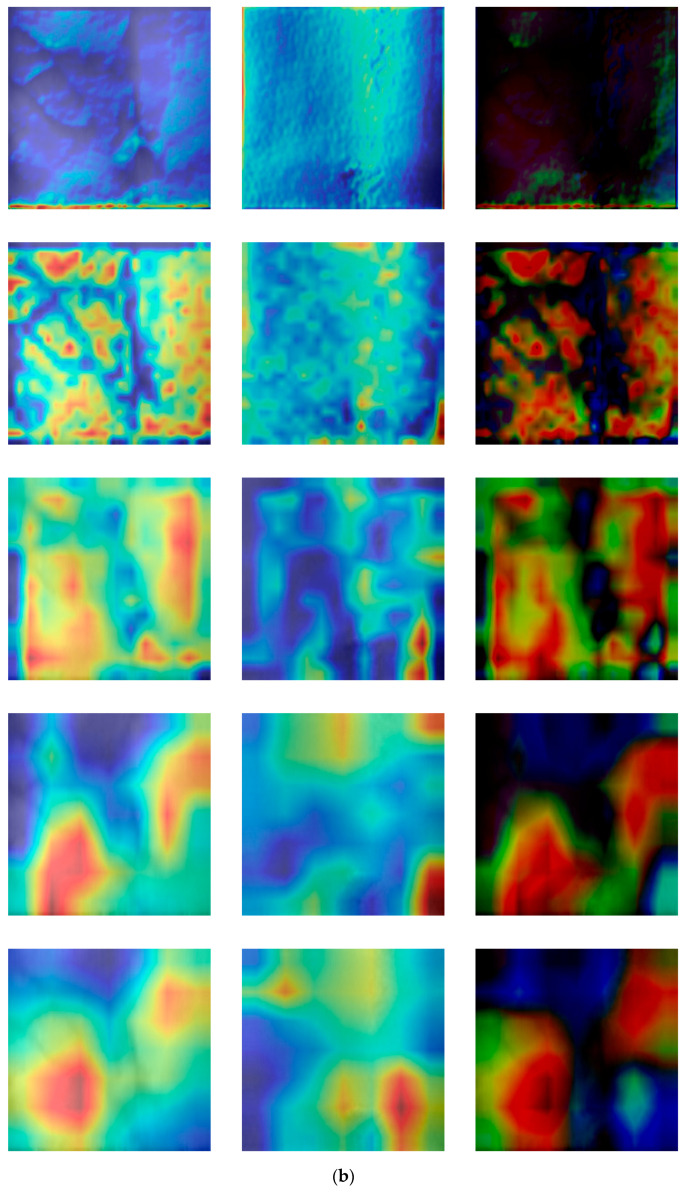
Comparisons of the class activation maps between the original and restored images. (**a**,**b**) are examples of authentic and imposter images, respectively. Images on the left of (**a**,**b**) are the original images, whereas those on the middle are the restored image by proposed modified DeblurGAN. In addition, the images on the right of (**a**,**b**) are the subtracted ones of the middle image from the left one. For both (**a**,**b**), the images from top to bottom are the class activation maps output from the 1st convolutional layer, the 1st transition layer, the 2nd transition layer, the 3rd transition layer, and the last dense block.

Figure 21 shows the result of visualizing each class activation map [56] based on the original images and those restored by the proposed modified DeblurGAN in each layer of DenseNet-161. The location from which the class activation map is output is the 1st convolutional layer, the 1st transition layer, the 2nd transition layer, the 3rd transition layer, and the last dense block layer from top to bottom. Figure 21a,b show examples of authentic (genuine) and imposter matching. The left and middle images in (a) and (b) are the original and restored images, respectively. Important features are represented in red, whereas insignificant features are represented in blue in the class activation map. Therefore, if the red and blue regions of the two images appear to be similar, it generally indicates that the two images have similar characteristics. As shown in Figure 21a, in authentic matching, class activation occurs in a similar location of the original image and restored image. Accordingly, it was confirmed that the motion blurred finger-vein image was effectively restored and correct acceptance is possible. As shown in Figure 21b, with imposter matching, class activation occurs in different locations in the original and restored image, implying that correct rejection is possible.

In addition, we included the subtracted CAM outputs of restored image from original (motion blurred) one in the right images of Figure 21a,b. The reasons of such differences in the subtracted CAM outputs are that the positions of important finger-vein features extracted are different in original and restored images. Nevertheless, the case of authentic matching (same class) shows the smaller differences as shown in the right image of the last row of Figure 21a compared to that of imposter matching (different classes) in the right image of the last row of Figure 21b. In addition, the reasons of such differences in the subtracted CAM outputs are that the important features of finger-vein can be newly extracted from the restored image (red color in the middle images of Figure 21a,b). However, they cannot be extracted from vein areas in original (motion blurred) image (red color in the left images of Figure 21a,b) due to the indistinctive vein patterns caused by motion blurring, but they are extracted from the other skin areas except for vein regions.

#### 4.8.2. Feature Maps of Difference Image

**Figure 22:**
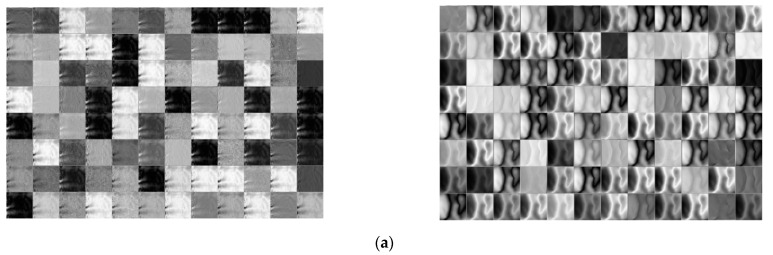

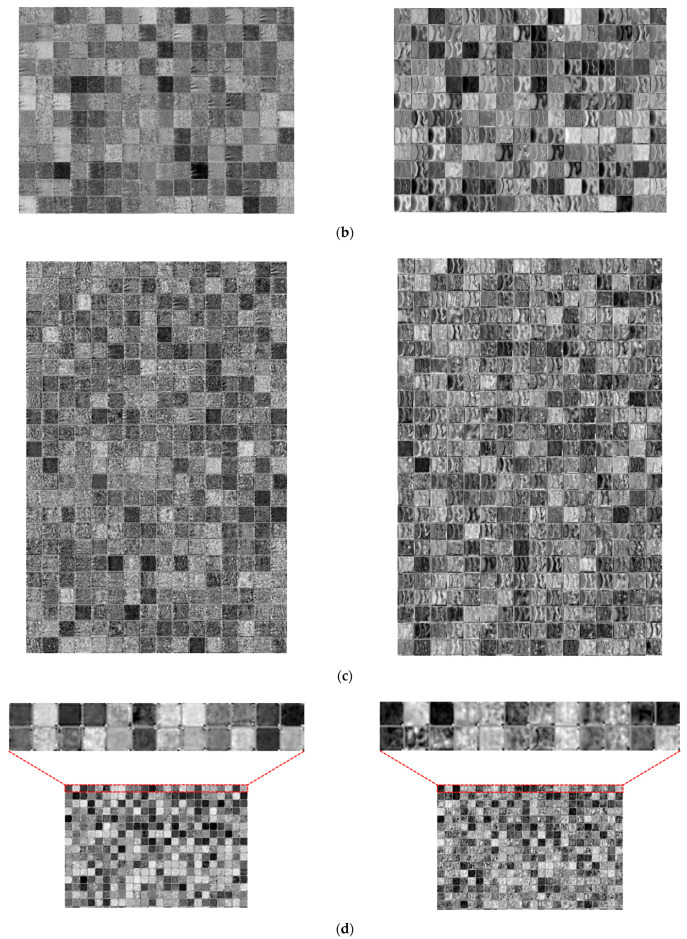

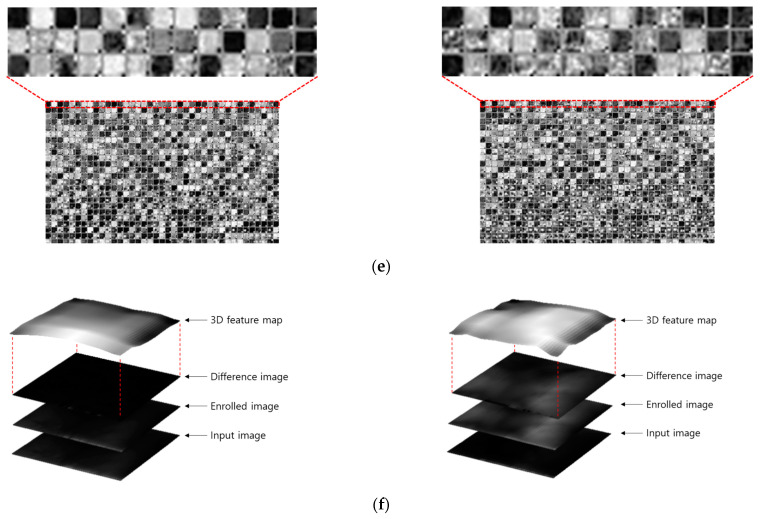
Feature maps extracted from a genuine matching image and an imposter matching image from several layers of the DenseNet-161. (**a**) Feature maps from the 1st convolutional layer; (**b**) feature maps from the 1st transition layer; (**c**) feature maps from the 2nd transition layer; (**d**) feature maps from the 3rd transition layer; (**e**) feature maps from the last dense block, and (**f**) 3D feature maps created by averaging feature map values of (**e**). Upper and lower examples in (**a**–**f**) represent genuine matching feature maps and imposter matching feature maps, respectively.

Second, similar to Figure 21, the feature maps of DenseNet-161 were analyzed according to the layer depth in which the difference image between the restored enrolled and restored recognized image as input. The input of DenseNet-161 is the finger-vein image restored by the modified DeblurGAN. As the feature map dimension is too large, the feature maps presented in Figure 22 are each channel’s output. Figure 22 presents the examples of the feature maps extracted from genuine and imposter matching images in several layers of DenseNet-161. Examples in Figure 22a–e are the feature maps extracted from the 1st convolutional layer, the 1st transition layer, the 2nd transition layer, the 3rd transition layer, and the last dense block, respectively. In addition, Figure 22f is the 3-dimensional feature map images created by averaging the feature map values of Figure 22e. The top and bottom images in Figure 22 show authentic and imposter matching, respectively.

As shown in Figure 22, abstract features were extracted as the layer became deeper. For example, low-level features, such as lines and corners of the original image, were maintained in Figure 22a, whereas, in Figure 22e, only the abstracted features remained, and shape information mostly disappeared. As shown in Figure 22a–e, the feature maps of authentic and imposter matching do not seem to have a significant difference. However, as shown in Figure 22f, although the changes in the 3-dimensional feature map values drawn by calculating the average of feature map values for the authentic matching results from a step before the classification layer were mostly flat, the results of imposter matching showed that the changes in the feature-map values were greater than those of authentic matching. Therefore, the difference in the CNN feature maps of authentic and imposter matching by the proposed method was confirmed.

## 5. Conclusions

In this study, a motion blurred finger-vein image was restored to solve the problem of deterioration of finger-vein recognition performance due to motion blur, and a recognition method using deep CNN was studied to evaluate the performance of the restored image. A modified DeblurGAN was proposed by modifying the original DeblurGAN, which was a restoration model. Using two open databases, the recognition error rate was lower when recognition was performed using the restoration method proposed in this study than when images were not restored. Furthermore, based on the comparative experiments using various state-of-the-art restoration models, the proposed method was more effective in restoring an image from motion blur and had more improved recognition performance. Also, based on the analysis of class activation maps and feature maps, it was confirmed that the proposed modified DeblurGAN sufficiently maintained the effective characteristics for classifying authentic and imposter matching. However, as mentioned in Figure 19, it was confirmed that incorrect matching cases occurred despite the proposed restoration method. Therefore, in future studies, a method of increasing restoration and recognition performance by overcoming the extreme difference in motion blur in intra-class and reducing the degree of similarity between inter-classes will be studied. In our research, we used the previous methods [27,29,30] for the ROI detection of finger region, and just focused on the restoration of motion-blur by our proposed modified DeblurGAN and finger-vein recognition by our CNN with the selected ROI. That is because the performance analysis is difficult if both the ROI detection and feature extraction of finger-vein are affected by motion blurring. Therefore, we assume that the ROI without motion blurring is correctly detected by the previous methods [27,29,30], and we only consider that the detected ROI is motion blurred. We would research the motion blurring effect on the boundary detection of ROI in future work.

If the enrolled and recognized images are captured from different camera settings, the performance of finger-vein recognition based on image difference can be affected. However, the enrolled and recognized images are captured from the same capturing device including same camera setting in usual cases of actual finger-vein recognition system. In addition, in this case, the recognition based on image difference showed the better accuracies than those based on original image with the extracted feature vector [20]. Therefore, we use this scheme of image difference for recognition because we mainly focused on the restoration of motion blurring by proposed modified DeblurGAN. We would research the recognition method with the enrolled and recognized images captured from different camera settings in future work.

People usually put their finger on the device with some guiding bar in the actual finger-vein acquisition device (with fixed finger direction) [29,31]. Therefore, there exist only the limited variations of the horizontal and vertical translation and in-plane rotation in the captured finger-vein image. Our data augmentation method aims at covering these individual variations, and it can reduce the recognition error (false rejection case). However, horizontal and vertical mirroring does not happen in the case of a finger-vein image acquisition of the actual capturing device. Therefore, the mirroring generates the images of different classes, which increases the complexity of training data and difficulties of model training. As shown in [57,58], singular value decomposition (SVD) can generate the images of various styles, which can also produce the images of different classes, and it can also increase the complexity of training data and difficulties of model training. Therefore, we use our simple data augmentation method. In future work, we would research the various data augmentation method including SVD and mirroring.

Also, the application of the proposed motion blur restoration method to other biometric modalities, such as iris, face, and palm-vein recognition, will be examined. Moreover, a lighter model that can shorten the processing time will be studied. In future work, we would also research the method with the cases of two open databases combined. In addition, as a future work, we would introduce different types of blurring to the images and develop a generic solution.

## Figures and Tables

**Table 1:** Comparisons of the previous and proposed finger-vein image restoration methods.

Category		Methods	Advantages	Disadvantages
Without considering blur restoration	Handcrafted feature-based	LBP-based feature extraction + Hamming distance [17]	Recognition performance is improved when an optimal filter is accurately modeled	- Performance degradation when the modeled optimal filter is applied to images having different characteristics - Not robust to image variants, such as illumination or misalignment, because the research was conducted in a constrained environment - A blur that may occur when capturing finger-vein images is not considered
		Gabor filter + SIFT feature matching [18]		
	Deep feature-based	PCA + LDA + SVM [19]	- No need to directly model an optimal filter - Robust to image variation as various image features are trained	- Requires intensive training process - Not consider a blur that may occur during image capturing
		Difference image + CNN [20,21]		
		Vein-pattern maps + CNN [22]		
		Composite image + shift matching + CNN [23,24,25]		
		SCNN-LSTM [26]		
Skin scattering blur restoration	Handcrafted feature-based	PSF + CLS filter [2]	Performance is significantly improved if scattering blur parameters are accurately estimated	- Scattering blur parameters must be accurately estimated - Parameters must be re-estimated when the domain between the image used for estimation and the test image is different
		BOM [3,4]		
		WBOM + ADAGC + NSTM + Gabor wavelets [5]		
		Haze removal techniques [6]		
		Multilayered PSF + BOM [7]		
		Optical model-based scattering removal [8]		
		Bilayer diffusion model + blur-SURE + multi-Wiener SURE-LET [9]		
Optical-blur restoration	Handcrafted feature-based	PSF for optical blur + PSF for scattering blur + CLS filter [10]	Image restoration considering both optical and skin scattering blur	- Performance can be improved only when the parameters of two PSFs are accurately predicted from the perspective of skin structure and camera optics - Processing time is long because optical blur restoration and skin scattering blur restoration are processed simultaneously
	Deep feature-based	Conditional GAN + CNN [11]	Applicable to images captured from various environments	Did not consider the motion blur
Motion blur restoration	Deep feature-based	Modified DeblurGAN-based method + CNN (Proposed method)	Recognition performance improved after restoration considering a motion blur that may occur when capturing finger-vein images	Networks for restoration and recognition require large data and take a long time to train.

**Table 2:** Descriptions of generator in modified DeblurGAN.

Layer		Number of Filters	Size of Feature Map (Height × Width × Channel)	Size of Kernel (Height × Width × Channel)	Number of Strides (Height × Width)	Number of Paddings (Height × Width)
Image input layer			256 × 256 × 3			
Encoder	1st convolutional layer Batch normalization ReLU	64	256 × 256 × 64	7 × 7 × 3	1 × 1	3 × 3
	2nd convolutional layer Batch normalization ReLU	128	128 × 128 × 128	3 × 3 × 64	2 × 2	1 × 1
	3rd convolutional layer Batch normalization ReLU	256	64 × 64 × 256	3 × 3 × 128	2 × 2	1 × 1
	Residual Blocks × 6 [3 × 3 conv, Batch normalization]	256	64 × 64 × 256	3 × 3 × 256	1 × 1	1 × 1
Decoder	1st transposed layer Batch normalization ReLU	128	128 × 128 × 128	3 × 3 × 256	2 × 2	
	2nd transposed layer Batch normalization ReLU	64	256 × 256 × 64	3 × 3 × 128	2 × 2	
	4th convolutional layer Batch normalization ReLU	3	256 × 256 × 3	7 × 7 × 64	1 × 1	3 × 3
	Output (input + 4th convolutional layer)		256 × 256 × 3			

**Table 3:** Descriptions of the discriminator in modified DeblurGAN (* means the output image or target image of Figure 5).

Layer	Number of Filters	Size of Feature Map (Height × Width × Channel)	Size of Kernel (Height × Width × Channel)	Number of Strides (Height × Width)	Number of Paddings (Height × Width)
* Image input layer		256 × 256 × 3			
1st convolutional layer Leaky ReLU	64	129 × 129 × 64	4 × 4 × 3	2 × 2	2 × 2
2nd convolutional layer Batch normalization Leaky ReLU	128	65 × 65 × 128	4 × 4 × 64	2 × 2	2 × 2
3rd convolutional layer Batch normalization Leaky ReLU	256	33 × 33 × 256	4 × 4 × 128	2 × 2	2 × 2
4th convolutional layer Batch normalization Leaky ReLU	512	34 × 34 × 512	4 × 4 × 256	1 × 1	2 × 2
5th convolutional layer	1	35 × 35 × 1	4 × 4 × 512	1 × 1	2 × 2

**Table 4:** Descriptions of experimental databases by data augmentation.

			SDU-DB	PolyU-DB
Original images		# of images	3816	1872
		# of people	106	156
		# of hands	2	1
		# of fingers	3 (index, middle, and ring fingers)	2 (index and middle fingers)
		# of classes (# of images per class)	636 (6)	312 (6)
Training for 1st or 2nd fold cross validation	Training of modified DeblurGAN	# of images (original + augmented data)	17,172 (6 images × 9 times × 318 classes)	8424 (6 images × 9 times × 156 classes)
	Training of CNN for finger-vein recognition	# of images for genuine matching	16,854 ((6 images × 9 times − 1) × 318 classes)	8268 ((6 images × 9 times − 1) × 156 classes)
		# of images for imposter matching	16,854 (Random selection)	8268 (Random selection)

**Table 5:** Comparison of finger-vein recognition error (EER) with respect to the applicable of a motion blur with SDU-DB (unit: %).

Training & Testing with Original Images (Scheme 1)	Testing Blurred Images without Training (Scheme 2)	Training & Testing with Blurred Images (Scheme 3)	Training & Testing with Restored Images (Scheme 4) (Proposed Method)
2.932	14.618	6.420	5.270

**Table 6:** Comparison of finger-vein recognition error (EER) with respect to the applicable of a motion blur with PolyU-DB (unit: %).

Training & Testing with Original Images (Scheme 1)	Testing Blurred Images without Training (Scheme 2)	Training & Testing with Blurred Images (Scheme 3)	Training & Testing with Restored Images (Scheme 4) (Proposed Method)
1.534	18.303	5.886	4.536

**Table 7:** Comparison of finger-vein recognition error (EER) of restored images in SDU-DB according to the perceptual loss based on the various CNN models and layers (unit: %).

VGG-19 [40] (Original DeblurGAN)	VGG-19 [40] (Conv3.1)	ResNeXt-101 [51] (Conv2)	ResNet-34 [32] (Conv2_x)
6.049	6.503	5.281	5.270

**Table 8:** Comparison of finger-vein recognition error (EER) of restored images in PolyU-DB according to the perceptual loss based on the various CNN models and layers (unit: %).

VGG-19 [40] (Original DeblurGAN)	VGG-19 [40] (Conv3.1)	ResNeXt-101 [51] (Conv2)	ResNet-34 [32] (Conv2_x)
4.777	4.536	4.764	4.983

**Table 9:** Comparisons of blur restoration by using the state-of-the-art methods and proposed modified DeblurGAN with PolyU-DB.

Methods	PSNR	SNR	SSIM
Original DeblurGAN [12]	28.98	21.45	0.90
DeblurGANv2 [14]	26.84	19.32	0.87
SRN-DeblurNet [15]	37.22	29.69	0.95
Modified DeblurGAN (proposed method) VGG-19 (conv3.1)	26.90	19.37	0.88
Modified DeblurGAN (proposed method) ResNet-34	27.70	20.17	0.90

**Table 10:** Comparisons of blur restoration by using the state-of-the-art methods and proposed modified DeblurGAN with SDU-DB.

Methods	PSNR	SNR	SSIM
Original DeblurGAN [12]	30.84	20.95	0.81
DeblurGANv2 [14]	29.63	19.73	0.82
SRN-DeblurNet [15]	39.17	29.28	0.90
Modified DeblurGAN (proposed method) VGG-19(conv3.1)	28.50	18.60	0.82
Modified DeblurGAN (proposed method) ResNet-34	32.64	22.75	0.85

**Table 11:** Comparisons of finger-vein recognition error (EER) by using the state-of-the-art restoration models and proposed methods with SDU-DB (unit: %).

Original DeblurGAN [12]	DeblurGANv2 [14]	SRN-DeblurNet [15]	Modified DeblurGAN
6.049	6.077	6.032	5.270

**Table 12:** Comparisons of finger-vein recognition error (EER) by using the state-of-the-art restoration models and proposed methods with PolyU-DB (unit: %).

Original DeblurGAN [12]	DeblurGANv2 [14]	SRN-DeblurNet [15]	Modified DeblurGAN
4.777	5.507	7.105	4.536

**Table 13:** Comparisons of processing speed by proposed method on desktop computer and embedded system (unit: ms).

	Modified DeblurGAN for Restoration	DenseNet-161 for Finger-Vein Recognition	Total
Desktop computer	3.4	12.8	16.2
Jetson TX2	6.1	226.2	232.3

## Data Availability

Not applicable.
